# Victims of Bullying: Emotion Recognition and Understanding

**DOI:** 10.3389/fpsyg.2021.729835

**Published:** 2021-10-14

**Authors:** Minita Franzen, Peter J. de Jong, Wim Veling, Marije aan het Rot

**Affiliations:** ^1^Department of Psychology, University of Groningen, Groningen, Netherlands; ^2^Department of Psychiatry, University Medical Centre Groningen, Groningen, Netherlands

**Keywords:** victims of bullying, emotion recognition, empathy, social behavior, interpersonal skills

## Abstract

**Introduction:** Victims of bullying often show interpersonal problems, such as having less high-quality interpersonal relationships compared to non-involved individuals. Research suggests that interpersonal struggles are associated with diminished emotional intelligence and competence and can lead to mental health problems such as depression. Therefore, we examined emotion recognition abilities, empathic accuracy, and behavioral responses to emotions in bullying victims and non-involved individuals. Based on previous research, we expected victims to show diminished skills in all three domains.

**Methods:** Adolescents (M_age_=17years; 67% female; no “other” gender participants) with (*N*=24) and without (*N*=21) a self-reported history of bullying victimization in high school completed a Virtual Reality facial emotion recognition task (ERT-VR), an empathic accuracy task (EAT) using videos of people recounting real-life autobiographical events, and a computer task in which they indicated their likely behavioral responses to facial emotions.

**Results:** The two groups only significantly differed in recognizing emotions when taking their depression symptoms into account. Across emotions, victims had lower recognition accuracy than non-involved individuals. When examining emotion-specific differences, victims showed lower accuracy for neutral faces which they mainly mistook for angry faces.

**Conclusion:** In contrast to expectations, adolescents with a high-school history of bullying victimization mostly showed similar emotional intelligence and competence skills as non-involved individuals. Nonetheless, we found some subtle differences regarding emotion recognition. Victims misjudged neutral as angry faces. This suggests a hostile attribution bias which might help explain victims’ interpersonal problems as well as their increased risk for mental health problems.

## Introduction

Bullying is conceptualized as an interpersonal act of systematic, repetitive, and intentional aggression toward someone who lacks power to self-defend (Olweus, 1994). For victims, it is a highly stressful and adverse experience. A factor that has been suggested both as precursor and consequence of victimization is dysfunctional interpersonal relationships. For example, compared to non-involved individuals, victims have more trouble forming and sustaining friendships and romantic relationships (e.g., Ellis and Zarbatany, 2007). Additionally, victims describe their interpersonal relationships as being of lower quality and lacking trust and affection (e.g., Goldbaum et al., 2003; Jantzer et al., 2006). Having such interpersonal struggles has also been suggested to play a role in explaining mental health problems such as depression, in bullied (Hansen et al., 2012; Arseneault, 2018), and non-bullied populations (Hammen, 2006; Beevers et al., 2007).

To better understand victims’ interpersonal struggles and thus also their mental health problems, researchers have recommended studying concepts such as emotional intelligence and competence (Mayer and Cobb, 2000; Lomas et al., 2012). Emotional intelligence represents the abilities to recognize and interpret one’s own and others’ emotions, along with using emotional information to guide and manage one’s own thoughts and emotional responses (Salovey and Mayer, 1990; Mayer et al., 1997, 2008). This also includes affective empathy (i.e., the ability to experience how another person feels) and cognitive empathy (i.e., the ability to understand how another person feels; see van Noorden et al., 2015). Emotional competence entails applying emotional intelligence skills to guide socially acceptable behavior (Zych et al., 2017). The appropriate utilization of emotional competencies is considered essential for successful social interactions and interpersonal relationships (Keltner and Haidt, 1999; Romasz et al., 2004). In the present study, our focus was on emotion recognition as well as understanding of other’s emotions.

Currently, there is mixed evidence regarding differences in emotion recognition and understanding emotional expressions between victims of bullying and non-involved individuals. Victims may have more difficulties with correctly recognizing and interpreting emotions. Lower emotion recognition has been prospectively related to a higher chance of experiencing peer victimization 6months later (Miller et al., 2005). Other cross-sectional studies have also reported victims being generally less accurate in recognizing facial emotional expressions, and specifically in recognizing anger, fear, and disgust compared to non-involved individuals (e.g., Ciucci et al., 2014). Victims have also been shown to overinterpret others’ intentions as hostile (Ziv et al., 2013) and to be prone to misclassify emotions such as fear as anger (Ciucci et al., 2014) but also to misclassify anger as fear (i.e., a fearful bias; cf. DiLalla and John, 2020). Of note however, most of these findings were qualified by type of victimization (Woods et al., 2009), the victim’s gender (Ciucci et al., 2014), or the intensity of the expressed emotion (Pozzoli et al., 2017). Additionally, one study found no evidence for emotion recognition difficulties in victims compared to bullies, bully-victims (i.e., persons who both bully others and are victimized), or non-involved individuals (Guy et al., 2017).

Results on the association between victimization and empathy are similarly inconsistent. In their systematic review of 40 studies, van Noorden et al. (2015) found victimization to be associated with reduced cognitive but not affective empathy, suggesting they are able to experience others’ feelings but not understand them well. Nonetheless, in a later empirical study, the same group found that perceived severity of victimization was positively associated with both types of empathy (van Noorden et al., 2016). Moreover, severe victims reported higher levels of both cognitive and affective empathy than non-involved individuals. While this could suggest that being victimized more severely might positively influence how victims understand and experience how others feel, the systematic review of van Noorden et al. (2015) suggests that being victimized might either not be related to empathic competencies, or that a negative association exists.

Emotional intelligence skills such as correctly identifying and interpreting emotions are fundamental to correctly process social information and to instigate appropriate interpersonal behaviors (cf. Social Information Processing Model; Crick and Dodge, 1996). Potential alterations in victims regarding emotion recognition and understanding, as described above, could therefore lead to altered interpersonal behaviors. In line with this, compared to non-involved individuals, victims have been reported to behave more submissively and lack assertiveness, while also reacting more aggressively (e.g., Perren and Alsaker, 2006; Lansford et al., 2010; Manring et al., 2018). These behaviors are generally perceived as dissatisfying and unpleasant by others (Moskowitz, 2009, 2010) and can perpetuate negative social interactions and bring about re-victimization (Dodge et al., 2003; Brendgen and Poulin, 2018). This in turn may explain why victims are more often rejected and have lower-quality social relationships than non-involved individuals (Ellis and Zarbatany, 2007; Veenstra et al., 2007). It seems therefore warranted to study multiple components of emotional intelligence and competence in victims simultaneously (Lansford et al., 2010; Lomas et al., 2012). This might help to further increase our understanding of victims’ interpersonal functioning.

Previous studies in victims mainly used non-immersive computer tasks showing static photographs of faces to assess emotion recognition (e.g., Woods et al., 2009; Ciucci et al., 2014), and self-report questionnaires to assess empathy (see van Noorden et al., 2016). Victims’ behaviors were often assessed through other-report (e.g., Perren and Alsaker, 2006; Lansford et al., 2010). In the present study, to better capture the dynamics and complexities of emotions and interpersonal situations (Fiorentini and Viviani, 2011; Hopwood et al., 2019), we examined aspects of emotional intelligence and competence using novel methodologies. Firstly, we employed a Virtual Reality (VR) emotion recognition task (ERT-VR). Compared to computer tasks showing morphed stimuli of gradually increasing emotional facial expressions on a screen (e.g., Pozzoli et al., 2017), VR offers increased ecological validity by creating a three-dimensional, immersive experience while keeping controlled laboratory conditions (Parsons, 2011; Gutiérrez-Maldonado et al., 2014). Secondly, in a separate empathic accuracy task (EAT), we asked participants to watch videos of people recounting real-life autobiographical events and rate how these targets felt. These ratings were then compared with the targets’ own ratings to create a measure of empathic accuracy (EA; considered a form of cognitive empathy) based on ecologically valid stimuli (cf. aan het Rot and Hogenelst, 2014). Finally, a third computer task was used to examine likely behavioral responses to facial emotions (aan het Rot et al., 2014). While this task was not designed with a focus on ecological validity and providing participants with an immersive experience, it does offer a controlled setting with set facial emotional expressions which enabled us to assess and compare potential interpersonal behaviors in social situations. In sum, by studying multiple components of emotional intelligence and competence using various and novel methods, we aimed to enhance knowledge about social-emotional skills of victims of bullying and to better understand victims’ interpersonal struggles.

### Hypotheses

We hypothesized that victims have overall a lower emotion recognition accuracy score during the VR task than non-involved participants (H1a). These differences in emotion recognition accuracy have also been suggested to be specific for anger, fear, and disgust (e.g., Ciucci et al., 2014). Therefore, we expected victims to have a lower accuracy for angry, fearful, and disgusted facial expressions (other emotions were not assessed) compared to their non-involved counterparts (H1b). Regarding empathy, we expected that victims would have less empathic accuracy compared to non-involved individuals (H2). As for responses to emotional facial expressions, we explored whether victims would generally show less agentic (i.e., less dominant and more submissive) responses (H3a). We also examined whether victims would show specifically low agency to angry and disgusted faces (H3b), because these facial expressions could serve as reminders of encounters with bullies.

## Materials and Methods

The study protocol was positively reviewed by the Ethics Committee of the Department of Psychology at the University of Groningen.

### Participants

#### Pre-screen

Participant recruitment involved contacting participants from a previous study on real-life interpersonal interactions of adolescents with and without a bullying history (Franzen et al., 2021), social media advertisements, and handing out flyers in the city of Groningen. The study was advertised as investigating interpersonal skills of adolescents with and without a history of bullying using VR. Adolescents of at least 16years of age could sign-up via an online questionnaire (*N*=78) where they were provided with detailed study information and provided active informed consent to participate in the pre-screen. They reported their contact details and completed the Olweus Bully/Victim Questionnaire (BVQ; Olweus, 1996, 2002) which assessed their bullying experiences during high school. Based on their answers on the BVQ, interested individuals were categorized as pure victims (*N*=30), bully-victims (*N*=12), pure bullies (*N*=5), and non-involved individuals (*N*=31; see materials and measures for details). Everyone but the pure bully group was invited to the main study.

#### Main Study

Of the 73 invited individuals, 46 chose to participate in the main part of the study. Financial compensation for study completion was 20€.

### Materials and Measures

#### Bullying Victimization

Bullying history during high school was assessed with an adjusted Dutch version of the BVQ (Lee and Cornell, 2009). The definition of bullying was a repeated, intentional act of aggression in a relationship where there is an imbalance of power. Participants stated to what extent they (A) were bullied by or (B) did bully others on a seven-point Likert scale ranging from 0=“Never,” 2=“Two or three times,” 4=“Two or three times per year,” to 6=“Several times per month.” Eight additional questions measured the frequency of (A) and (B) regarding physical, verbal, social, sexual, and electronic bullying, or because of body weight, race or religion, and disability.

Participants were categorized as *pure victims* if they scored 2 or higher on any of the (A) questions and below 2 on all of the (B) questions. Participants were categorized as *bully-victims* if they scored 2 or higher on any of the (A) questions as well as on any of the (B) questions. A *pure bully* scored 2 or higher on any of the (B) but below 2 on all of the (A) questions. A *non-involved* individual scored 0 or 1 on both (A) and (B) questions.

#### Emotion Recognition Task

Emotion recognition abilities were tested in a virtual reality (VR) environment using the ERT-VR created by CleVR (Delft, The Netherlands) with Unity software. The ERT-VR is part of a VR training module for social cognition training (i.e., DiSCoVR; see Nijman et al., 2019 for details). Participants were presented a VR 3D shopping mall through a head mounted display (Oculus DK2; Rift development kit 2) with a resolution of 1,080×960 per eye. Random “shopping mall background noises” were played throughout the task. By operating a joystick (either Xbox 360 or Nintendo SNES), participants moved through the VR environment. Avatars were standing (*N*=27) or walking around (*N*=8) within the VR shopping mall. Similar to the distribution of ethnicities in the Northern Netherlands, avatars mainly had a Caucasian appearance (92%; Centraal Bureau voor de Statistiek, 2016).

Participants were instructed to approach all standing avatars and to indicate as quickly and precisely as possible which emotion they saw in the avatar’s face. Once an avatar was approached (i.e., when the participant moved within a 2m radius within the VR environment), it turned toward the participant and randomly displayed a dynamic facial emotion up to a specified intensity of either 50 or 75%, or a neutral facial expression. In total, it was planned to show four angry, disgusted, and fearful faces for each intensity (i.e., 24) plus four neutral facial expressions (i.e., 28 in total). Due to a systematic programming error, only 27 avatars were shown to all participants resulting in one of the possible 28 facial expressions missing at random. While an avatar displayed an emotion, the question “Which emotion?” was presented together with four answer options (i.e., angry, disgusted, fearful, and neutral) to the right of the avatar. Participants had one attempt and 30s to answer the question before the avatar would walk away. The correct answer lit up in green while an incorrect answer was displayed in red. This feedback feature was non-adjustable. Once all 27 avatars were approached, the VR environment stopped automatically.

Accuracy scores per emotion (disregarding intensity) were created by dividing all correct responses for the particular emotion by the number of possible correct answers which was either 7 or 8 due to the programming error. The accuracy score for neutral facial expressions were calculated similarly, again adjusting for the maximum of answer options of 3 or 4. Analogously, we also created accuracy scores per emotion that took intensity (either 50 or 75%) into account. The number of correct responses per emotion and intensity was divided by the number of possible correct answers (i.e., 3 or 4).

#### Empathic Accuracy Task-VR

Empathic accuracy was measured using a shortened version of the task developed by aan het Rot and Hogenelst (2014). Similar to Elzinga et al. (2018), a total of 10 of the original 20 validated video clips were used to keep the total assessment battery under 60min. Video clips consisted of five female and five male targets recounting positive (e.g., being happy about being accepted into a student association, or friends organizing a surprise birthday party) or negative (e.g., being sad about the end of a relationship or a friend’s unexpected death) personal experiences. Participants were presented with videos in a semi-random order, meaning that there were never more than two positive or negative videos and never the same target in a row. On a dial which corresponded to a nine-point Likert scale, anchored from 1 (extremely negative), over 5 (neutral), to 9 (extremely positive), participants continuously rated how targets felt. The continuous rating data were averaged across 5-s intervals. The first and final 5s of all ratings were discarded. In line with aan het Rot and Hogenelst (2014), we transformed the data using the Yule-Walker method. Similar to previous studies applying the EAT, an empathic accuracy score was calculated for each participant/clip combination by correlating participants’ ratings to the target’s own ratings (who rated their own videos in the same manner as the present participants) using Pearson correlations. The targets previously also rated their own level of expressivity with the self-report Berkeley Expressivity Questionnaire (BEQ; Gross and John, 1997). Expressivity was assessed because between-target differences in expressivity can influence perceiver empathic accuracy (Zaki et al., 2008).

#### Facial Emotional Response Task

Similar to aan het Rot et al. (2021), we assessed participants’ responses to facial emotions using an adapted version of the task developed by aan het Rot et al. (2014). Stimuli of facial expressions consisted of grayscale faces of six male and six female persons who displayed emotional faces (i.e., angry, disgusted, and happy) or a neutral face. Emotional faces were presented at 50 and 100% intensity. The 84 faces were randomly presented on a screen for 500ms each. In between each face, a fixation symbol was shown for 300ms. Following each face, participants were asked to rate how they would likely behave toward the person they just saw. Ratings were given by clicking a mouse cursor on an interpersonal grid (cf. Moskowitz and Zuroff, 2005). The horizontal axis ranged from quarrelsome (left, score of −100) to agreeable (right, score of +100) behavior representing communion. The vertical axis ranged from dominant (top, score of +100) to submissive (bottom, score of −100) behavior representing agency. Clicking the center of the grid resulted in a score of 0 for both axes representing neutral behavior. Participants were given a response time of 5,000ms which was indicated by a time bar displayed above the grid. Mean scores on communion and agency were significantly yet weakly correlated (*r*=0.08, *p*<0.0001).

#### Depression Symptoms

Depression symptoms in the previous week were assessed with the 21-item Depression Anxiety and Stress Scale (DASS-21; Lovibond and Lovibond, 1995; de Beurs et al., 2001). Participants indicated to what degree they had experienced symptoms on a four-point Likert scale ranging from 0 (not at all/never) to 3 (very much/most of the time). The total score of the seven-item depression subscale was doubled to fit cut-off scores of the original 42-item DASS and therefore ranged from 0 to 42. The Cronbach’s coefficient alpha was 0.93 indicating excellent internal consistency.

#### Social Anxiety

We assessed two forms of social anxiety. Using the Social Interaction Anxiety Scale (SIAS; Mattick and Clarke, 1998; 20 items), we assessed behavioral and emotional aspects of social anxiety. Cognitive features of social anxiety were assessed with the Brief Fear of Negative Evaluation scale (BFNE; Leary, 1983; 12 items). Participants were asked to indicate to what extent each item is characteristic of them on a five-point Likert scale ranging from 0 (not at all) to 4 (extremely). An example of the SIAS is “I become tense if I have to talk about myself or my feelings.” The BFNE includes items such as “I am usually worried about what kind of impression I make.” For both questionnaires, items were added up resulting in a maximum score of 80 for the SIAS and 48 for the BFNE. Internal consistency was excellent for both SIAS (Cronbach’s alpha=0.92) and BFNE (Cronbach’s alpha=0.96).

#### Dizziness, Nausea, and Headaches

Participants can experience cyber sickness during VR tasks (Kennedy et al., 1993). Therefore, we assessed dizziness, nausea, and headache pre- and post-ERT-VR with visual analogue scales (VAS) ranging from 0 to 100.

### Procedure

For the main part of the study, participants were given a printed study information sheet and gave written informed consent for the second time. Right before the respective tasks, they were asked to fill in online questionnaires as indicated. Afterwards, participants performed the tasks in the following order: ERT-VR, EAT, and Facial Emotional Response Task (FERT). Before each task, participants were given detailed instructions and were able to practice the task at hand. The practice trial for the ERT-VR consisted of avatars showing 100% happy faces to avoid a learning effect. Before and after each task, participants filled in the VAS. Participants were given the chance of a break including a beverage and a small snack between the tasks.

### Data Analyses

The initial number of participants was 46 (i.e., N_victims_=24, N_non-involved_=21, and N_bully-victim_=1). To ensure roughly equal group sizes for between-group comparisons, we excluded the participant with the bully-victim status from final analyses. Other reasons for exclusion from final analyses were: Participation in a pilot study that included the ERT-VR, missing data due to failure of equipment, or due to failure of the computer task. This resulted in slightly different group sizes per task (i.e., N_ERT-VR_=37, N_EAT_=40, and N_FERT_=43). See Table 1 for more details.

**Table 1:** Descriptive statistics for participant characteristics, mental health, and the ERT-VR and EAT.

	Victims	Non-involved	Total
N of full completers (% female^A^)	24 (63)	21 (76)	45 (67)
Final *N* as used in analyses			
ERT-VR	21	16	37
EAT	20	20	40
FERT	23	21	43
Age range in years	16–19	16–19	16–19
Mean age in years (SD)	16.79 (0.93)	16.86 (0.86)	16.82 (0.89)
Mean depression symptoms (SD)	17.75 (13.23)	6.76 (4.75)	12.62 (11.54)
Mean social anxiety symptoms (SD)			
SIAS	28.29 (14.71)	16.76 (7.51)	22.91 (13.14)
BFNE	25.17 (13.24)	16.10 (10.42)	20.93 (12.72)
Mean dizziness (SD)			
Pre-ERT-VR	4.54 (7.77)	1.32 (3.97)	3.05 (6.46)
Post-ERT-VR	11.46 (13.53)	6.71 (8.39)	9.04 (11.50)
Mean nausea (SD)			
Pre-ERT-VR	2.04 (5.61)	6.42 (22.95)	3.89 (15.57)
Post-ERT-VR	6.29 (11.61)	4.62 (7.08)	5.39 (9.62)
Mean headache (SD)			
Pre-ERT-VR	6.88 (10.08)	4.21 (9.87)	5.57 (9.88)
Post-ERT-VR	8.58 (10.31)	3.86 (6.57)	6.24 (8.94)
ERT-VR			
Mean emotion recognition accuracy (SD)			
Overall	0.61 (0.14)	0.70 (0.08)	0.64 (0.12)
Angry	0.62 (0.18)	0.58 (0.17)	0.61 (0.18)
Disgusted	0.38 (0.25)	0.45 (0.19)	0.41 (0.22)
Fearful	0.73 (0.17)	0.85 (0.14)	0.78 (0.16)
Neutral	0.72 (0.27)	0.91 (0.14)	0.80 (0.24)
EAT			
Mean empathic accuracy (SD)			
Overall	0.16 (0.24)	0.11 (0.26)	0.14 (0.25)
For positive videos	0.37 (0.33)	0.31 (0.31)	0.34 (0.32)
For negative videos	−0.05 (0.33)	−0.08 (0.28)	−0.07 (0.30)

A While the option “other” was provided when assessing gender, no participant endorsed it. ERT-VR, emotion recognition task – virtual reality; EAT, empathic accuracy task; FERT, facial emotional response task; SIAS, social interaction anxiety scale; and BFNE, brief fear of negative evaluation scale. Descriptive statistics for participant characteristics and mental health are based on all study completers. Descriptive statistics for the ERT-VR and EAT are based on respective number of participants who completed the task. Cut-off scores for depression symptoms: mild (10–13), moderate (14–20), severe, or extremely severe (21+). Cut-off score for SIAS: >42 clinically significant social anxiety. Cut-off score for BFNE: >24 clinically significant social anxiety.

All analyses were performed in SAS version 9.4 (SAS Institute: Cary, NC). The level of significance was set at an alpha of 0.05. Effect sizes for significant effects are expressed using Cohen’s d. Group mean comparisons for depression symptoms and social anxiety were performed using independent sample t-tests. Differences in pre- and post-scores for dizziness, nausea, and headaches by victimization status were tested with repeated measures analyses of variances.

All main analyses for all three tasks (i.e., ERT-VR, EAT, and FERT) were performed in SAS using PROC MIXED with maximum likelihood estimation. Fixed effects or between-person means (i.e., victims and non-involved) were compared based on least squares means (LS-means) with Tukey correction for multiple comparison.

For all main analyses of the ERT-VR, the data were treated as repeated measures within-person per emotion (i.e., neutral, angry, disgusted, fearful) and intensity of emotion (50 or 75%). Two main models were tested, with emotion-accuracy as dependent variable. Model 1 tested the effect of victimization status on overall accuracy, across emotions (i.e., victimization status as single predictor). The second model tested the effect of victimization status on emotion accuracy per type of emotion (i.e., adding the interaction term of victimization status by type of emotion). Originally, we also tested a third model in which we considered intensity of emotion as an additional within-subjects factor (i.e., a three-way interaction of victimization status by type of emotion by intensity of emotion) which yielded non-significant results. As we are aware of the low statistical power of the current study to find small effects of such complex associations, we decided against reporting results of the third model.

We added depression as a covariate in the final models based on literature suggesting that depression scores can be negatively associated with emotion recognition accuracy (for a review, see Dalili et al., 2015); victims in our sample had significantly higher depression symptoms than non-involved peers (see Table 1). We report results of models with and without depression symptoms due to different outcomes regarding significance level.

For main analyses of the EAT, original empathic accuracy scores based on correlations between perceiver and target ratings were transformed to Fisher *z* scores prior to analyses. Final models included perceiver and target as random effects. The first model tested the effect of victimization status (victim vs. non-involved) on empathic accuracy (i.e., victimization status as single predictor). In subsequent models, we examined whether the effect of victimization status was moderated by valence of the video clips (positive or negative) or by expressivity of the targets.

We also tested whether target gender (i.e., of individuals in the videos clips), perceiver gender (i.e., of participants), or depression scores should be added as covariates to the final models by testing them as separate single predictors of empathic accuracy. Based on significant results, depression and target gender but not perceiver gender were added as covariates to the final models.

Main FERT analyses consisted of two models. Model 1 tested a main effect of victimization status (victim vs. non-involved) on agentic and communal behavioral responses (i.e., victimization status as single predictor). Model 2 included the interaction effect of victimization status by emotion (neutral, angry, disgusted, and happy) as predictor of either agentic or communal behavioral responses. We initially also tested a third model, which included intensity of emotion (50 or 100%) as an additional within-subjects factor (i.e., a three-way interaction of victimization status by type of emotion by intensity of emotion). Results were non-significant. For similar reasons as explained above for results of model 3 of the ERT-VR, we decided to not report results of the third model of the FERT.

Similar to previous studies who reported varying response times by facial expression and by target (e.g., participants generally taking longer to respond to angry compared to happy faces; aan het Rot et al., 2017), we tested emotion expression as predictor of response time (square-root transformed due to skewness). Based on significant effects, we added response time as a covariate in the final models. Additionally, we added depression as a covariate to the final models due to literature reporting depression to be associated with alterations in agency and communion (Painuly et al., 2005; Hames et al., 2013).

## Results

**Figure 1:**
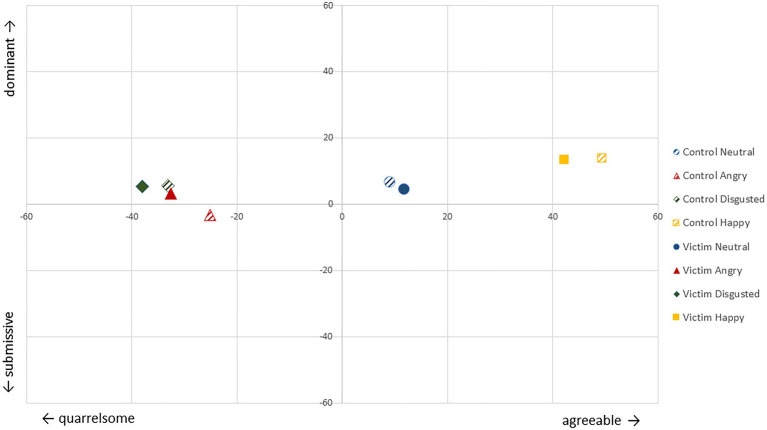
Likely communal (quarrelsome-agreeable) and agentic (submissive-dominant) behavioral responses of victims and non-involved individuals per emotion.

Detailed information on descriptive statistics for participant characteristics, mental health variables, and the ERT-VR and EAT can be found in Table 1; for FERT see Figure 1. Pearson’s correlations between study variables can be found in Table 2.

**Table 2:** Between-person Pearson’s correlations for study variables and descriptive statistics.

Variables	1	2	3	4	5	6	7	8	9	10	11
1. Emotion accuracy overall	-										
2. Emotion accuracy anger	**0.53**	-									
3. Emotion accuracy disgust	**0.71**	0.17	-								
4. Emotion accuracy afraid	**0.60**	−0.02	0.16	-							
5. Emotion accuracy neutral	**0.52**	0.04	0.11	**0.39**	-						
6. Empathic accuracy	0.29	0.27	0.07	0.16	0.23	-					
7. Communal behavior	0.22	−0.22	0.21	**0.37**	0.13	0.11	-				
8. Agentic behavior	0.25	0.12	0.15	0.22	−0.03	−0.23	0.09	-			
9. Depression	0.03	0.24	−0.16	−0.21	0.30	**0.40**	−0.14	−0.07	-		
10. Social anxiety –BFNE	**−0.30**	0.08	**−0.38**	**−0.30**	−0.03	0.18	−0.01	−0.24	**0.38**	-	
11. Social anxiety – SIAS	**−0.44**	−0.04	**−0.42**	**−0.45**	−0.05	0.26	−0.08	**−0.40**	**0.56**	**0.73**	-
Mean	0.63	0.61	0.41	0.78	0.8	0.14	−3.75	6.68	12.62	20.93	22.91
*SD*	0.12	0.18	0.22	0.16	0.24	0.25	17.1	17.75	11.54	12.72	13.14

Values significant at an alpha of 0.05 are represented in bold. SIAS, social interaction anxiety scale; BFNE, brief fear of negative evaluation scale.

### Descriptive Statistics and Mental Health

The majority of victims (63%) reported having been bullied once a month or more during high school. Another 18% indicated victimization experiences two to three times a year and the other 19% two to three times during high school. Therefore, our sample experienced moderate to severe victimization.

Victims reported significantly more depression symptoms than non-involved peers [*t*(43)=−3.59, *p*<0.001]. Victims also reported significantly more social anxiety symptoms than non-involved participants, based on both the SIAS [*t*(43)=−3.24, *p*=0.002] and the BFNE [*t*(43)=−2.53, *p*=0.02].

Victims and non-involved individuals had low mean scores of dizziness, nausea, and headache before and after the ERT-VR. Pre- and post-ERT-VR group means did not differ significantly, neither for dizziness [*F*(1,41)=0.35, *p*=0.56], nausea [*F*(1,41)=1.27, *p*=0.27], nor headache [*F*(1,41)=1.29, *p*=0.26].

### Emotion Recognition Accuracy

For detailed test statistics, please refer to Table 3.

**Table 3:** Associations between victimization status and emotion recognition accuracy and moderation effects of type of emotion.

Predictors	Outcome	*F*	*p*
*Model 1a*	Emotion recognition accuracy		
Victim status		3.76	0.061
*Model 1b*			
Victim status		**5.61**	**0.024**
Depression symptoms		2.07	0.159
*Model 2a*			
Victim status		**4.72**	**0.037**
Type of emotion		**36.55**	**<0.0001**
*Model 2b*			
Victim status		**4.75**	**0.036**
Type of emotion		**39.76**	**<0.0001**
Victim status*Type of emotion		2.44	0.069
*Model 2c*			
Victim status		**7.61**	**0.009**
Type of emotion		**36.58**	**<0.0001**
Depression symptoms		2.81	0.103
*Model 2d*			
Victim status		**7.59**	**0.009**
Type of emotion		**39.80**	**<0.0001**
Depression symptoms		2.87	0.101
Victim status*Type of emotion		2.46	0.067

Values significant at an alpha of 0.05 are represented in bold.

#### Group Difference in Overall Accuracy (H1a)

The covariate analysis suggested a significant difference between victims and non-involved participants (*d*=0.81; see model 1b in Table 3). Victims had a lower overall emotion recognition accuracy (*M*=0.60, *SE*=0.03) than non-involved peers (*M*=0.71, *SE*=0.03). In the model without depression symptoms as covariate, this difference was not significant according to the predetermined alpha (*d*=0.66; see model 1a in Table 3). However, the same trend was observed.

#### Group Differences in Recognition Accuracy for Specific Emotions (H1b)

In the covariate analysis, the emotion by group interaction for accuracy was not significant (*d*=0.31; see model 2d in Table 3). However, as the value of *p* was 0.067, we continued to examine *post hoc* emotion by group comparisons. One relevant group by emotion comparison was significant, namely for neutral faces, which remained significant after Tukey-Kramer correction [*t*(105)=3.19; *p*=0.039; *d*=0.62]. More specifically, victims (*M*=0.71, *SE*=0.04) had a lower accuracy for neutral faces than non-involved individuals (*M*=0.92, *SE*=0.05). On average, victims mislabeled neutral faces 29% of the time (or 22 times across the entire task) while non-involved individuals mislabeled them 8% of the time (or four times across the entire task). If they made a mistake, victims mistook neutral as angry faces 68% (15/22 times) of the time, as disgusted faces 27% (6/22 times) of the time, and as fearful 5% (1/22 times) of the time. In comparison, non-involved individuals either mistook neutral as angry faces, namely 75% (or 3/4 times) of the time, or as fearful, 25% (1/4 times) of the time.

We also tested for group differences in misattribution for neutral faces. Focusing on angry-misattribution, the group difference approached significance [*t*(35)=−1.89; *p*=0.067; *d*=0.64], with victims having a higher mean (*M*=0.46, *SD*=0.47) for misattributing neutral faces compared to non-involved individuals (*M*=0.19 *SD*=0.40). As for fearful-misattribution, there was no significant group difference [*t*(35)=0.58; *p*=0.569; *d*=0.20]. No group comparison for disgust was possible as the non-involved group did not have any disgust-misattribution.

In the model without depression symptoms as covariate, type of emotion did not significantly moderate the association between victimization and emotion accuracy (*d*=0.30; see model 2b in Table 3). While no relevant group by emotion comparisons were significant after Tukey-Kramer correction, there was a trend observed for the group difference for neutral faces [*t*(105)=2.83; *p*=0.099; *d*=0.55].

### Empathic Accuracy

For detailed test statistics, please refer to Table 4.

**Table 4:** Associations between victimization status and empathic accuracy and moderation effects of video clip valence and of targets’ expressivity.

Predictors	Outcome	*F*	*p*
*Model 1a*	Empathic accuracy		
Victim status		0.43	0.514
*Model 1b*			
Victim status		0.47	0.499
Depression symptoms		**7.72**	**0.009**
Target gender		3.58	0.066
*Model 2a*			
Victim status		0.42	0.522
Valence		**47.10**	**<0.0001**
*Model 2b*			
Victim status		0.42	0.522
Valence		**47.14**	**<0.0001**
Victim status*Valence		0.07	0.789
*Model 2c*			
Victim status		0.48	0.492
Valence		**43.60**	**<0.0001**
Depression symptoms		**7.72**	**0.009**
Target gender		0.26	0.614
*Model 2d*			
Victim status		0.48	0.491
Valence		**43.64**	**<0.0001**
Depression symptoms		**7.72**	**0.009**
Target gender		0.26	0.614
Victim status*Valence		0.07	0.789
*Model 2e*			
Victim status		0.44	0.513
Expressivity		0.95	0.329
*Model 2f*			
Victim status		0.46	0.504
Expressivity		0.95	0.331
Victim status*Expressivity		0.29	0.591
*Model 2g*			
Victim status		0.47	0.499
Expressivity		0.02	0.876
Depression symptoms		**7.72**	**0.009**
Target gender		2.65	0.112
*Model 2h*			
Victim status		0.13	0.719
Expressivity		0.02	0.875
Depression symptoms		**7.72**	**0.009**
Target gender		2.66	0.111
Victim status*Expressivity		0.29	0.588

Values significant at an alpha of 0.05 are represented in bold.

#### Group Difference in Overall Empathic Accuracy (H2)

There was no significant difference between victims and non-involved peers in empathic accuracy, neither in the covariate analysis (*d*=0.24; see model 1b in Table 4) nor in the model without covariates (*d*=0.21; see model 1a in Table 4).

#### The Role of Clip Valence and Target Expressivity (Exploratory)

There was no significant moderation effect of video clip valence on the association between victimization and empathic accuracy; neither in the covariate analysis (*d*=0.09; see model 2d in Table 4) nor in the model without covariates (*d*=0.09; see model 2b in Table 4). Notably, both victims and non-involved peers performed particularly poorly when viewing the negative video clips (for means see Table 1).

There was also no moderation effect of target expressivity on the association between victimization and empathic accuracy; neither in the covariate analysis (*d*=0.08; see model 2h in Table 4) nor in the model without covariates (*d*=0.06; see model 2f in Table 4).

### Behavioral Responses to Facial Emotions

Please refer to Table 5 for detailed statistics.

**Table 5:** Associations between victimization status and agentic and communal behavior and moderation effects of type of emotion.

Predictors	Outcome	*F*	*p*
*Models 1a*			
Victim status	Agentic behavior	0.06	0.811
Victim status	Communal behavior	1.24	0.272
*Models 1b*			
Victim status		0.27	0.605
Response time	Agentic behavior	0.86	0.354
Depression symptoms		0.43	0.514
			
Victim status		0.59	0.446
Response time	Communal behavior	3.32	0.069
Depression symptoms		0.22	0.641
			
*Models 2a*			
Victim status	Agentic behavior	0.06	0.807
Emotion		**22.38**	**<0.0001**
Victim status	Communal behavior	1.12	0.296
Emotion		**144.78**	**<0.0001**
			
*Models 2b*			
Victim status		0.03	0.872
Emotion	Agentic behavior	**22.58**	**<0.0001**
Victim status*emotion		2.26	0.085
			
Victim status		0.77	0.386
Emotion	Communal behavior	**146.54**	**<0.0001**
Victim status*emotion		2.26	0.085
			
*Models 2c*			
Victim status		0.29	0.596
Emotion	Agentic behavior	**22.11**	**<0.0001**
Response time		0.05	0.825
Depression symptoms		0.44	0.512
			
Victim status		0.43	0.514
Emotion	Communal behavior	**149.35**	**<0.0001**
Response time		**10.79**	**0.001**
Depression symptoms		0.25	0.622
			
			
*Models 2d*			
Victim status		0.21	0.649
Emotion	Agentic behavior	**22.30**	**<0.0001**
Victim status*emotion		2.26	0.085
Response time		0.05	0.820
Depression symptoms		0.44	0.513
			
Victim status		0.24	0.626
Emotion	Communal behavior	**151.53**	**<0.0001**
Victim status*emotion		2.42	0.070
Response time		**11.26**	**0.001**
Depression symptoms		0.25	0.621

Values significant at an alpha of 0.05 are represented in bold.

#### Group Differences in Agentic Behavior (H3a) and Communal Behavior (Exploratory)

There was no significant difference between victims and non-involved participants in agentic behavior; neither in the covariate analysis (*d*=0.16; see model 1b in Table 5) nor in the model without covariates (*d*=0.08; see model 1a in Table 5). There was also no significant group difference in communal behavior; neither in the model including covariates (*d*=0.24; see model 1b in Table 5) nor in the model without covariates (*d*=0.35; see model 1a in Table 5).

#### Group Differences in Agentic Behavior by Specific Emotions (H3b) and in Communal Behavior by Specific Emotions (Exploratory)

There was no overall moderation effect of emotion on the association between victimization and agentic behavior; neither in the covariate analysis (*d*=0.27; see model 2d in Table 5) nor in the model without covariates (*d*=0.27; see model 2b in Table 5). No relevant group by emotion comparisons was significant after Tukey-Kramer correction. Similarly, emotion did also not significantly moderate the association between victimization and communal behavior; again, neither in the model including covariates (*d*=0.28; see model 2d in Table 5) nor in the model without covariates (*d*=0.27; see model 2b in Table 5). No relevant group by emotion comparisons was significant after Tukey-Kramer correction.

### Summary of Results

Our first hypothesis (H1a) was supported when (as planned) depression symptoms were included as a covariate in the analysis, and the same trend was seen when this covariate was not included. Victims had a lower overall emotion recognition accuracy than non-involved peers. Our hypothesis regarding less accuracy in victims for angry, fearful, or disgusted faces compared to non-involved participants (H1b) was not supported. Rather, compared to their non-involved peers, victims had more problems identifying neutral faces. Both groups mainly mistook neutral faces as angry and means for this misattribution suggested victims to have had more problems with that. However, this group difference for angry-misattribution only approached significance. We also expected victims to be worse in rating how other people feel (i.e., empathic accuracy, EA). However, the most salient result for the EA task was that our sample generally showed poor EA compared to previous research (aan het Rot and Hogenelst, 2014; Hogenelst et al., 2016; Thiel et al., 2018). When examining behavioral responses to facial expressions, we found that participants indicated being agreeable toward others with happy expressions, and quarrelsome toward angry and disgusted expressions (see Figure 1). However, results did not support our hypotheses that victims would show less agentic (i.e., more submissive) responses overall (H3a) or when specifically seeing angry and disgusted faces (H3b) compared to non-involved individuals.

## Discussion

In the present study, we examined aspects of emotional intelligence and emotional competence in teenagers with and without bullying victimization experiences. We found some subtle differences regarding emotion recognition accuracy when taking depression symptoms into account and no significant differences in empathic accuracy or behavioral responses to facial emotions. Thus, in contrast to what we expected, victims mostly showed similar social-emotional skills compared to non-involved individuals.

Victims have been reported to have interpersonal struggles (e.g., Ellis and Zarbatany, 2007). As a potential reason, some research suggests alterations in victims’ emotional intelligence and competence. For example, victims have been reported to be less accurate in recognizing emotions (e.g., Pozzoli et al., 2017). In line with this, we found that victims had more trouble recognizing emotions and specifically neutral faces compared to their non-involved peers. Angry-misattribution (i.e., labeling a neutral face as angry) appeared more frequent for victims, although group differences only approached significance. Nevertheless, overall, victims were statistically less accurate in recognizing neutral faces. This suggests that victimized individuals had a biased interpretation of social information. Specifically, they appeared to attribute hostile intentions to others. Such a hostile attribution bias could stem from adverse experiences with bullies that generalize to other situations. Victims’ increased social anxiety scores (compared to non-involved peers) also point toward them fearing or expecting to be negatively judged by others. Having such negative expectations and perceptions of others can influence the quality of social relationships (Guy et al., 2017) and has also been found to be associated with depression symptoms (Gadassi and Rafaeli, 2015; Belmans et al., 2019).

Of note, we did find these differences in emotion recognition between victims and non-involved individuals when considering underlying depression symptoms but not when we tested the association without taking depression symptoms into account. Victims in our sample, similar to previous research (e.g., Moore et al., 2017), had significantly more depression symptoms than non-involved individuals. Therefore, and due to research reporting depression symptoms to influence interpersonal skills such as emotion recognition (e.g., Dalili et al., 2015), we chose to include it in our final analyses. Our significant results suggest a negative relationship between bullying victimization and emotion recognition accuracy independent of underlying depression symptoms. The fact that, although pointing in the same direction, results did not reach significance when disregarding depression symptomology, could be explained by our small group sizes and therefore low statistical power. While there are no standard power analyses available for multilevel models, deducing from the Cohen’s *d*s of our significant effect (*d*=0.81) compared to the non-significant effects (*d*s<0.67), we assume that we had enough power to detect large but likely lacked power to detect medium and small effects. However, we would like to highlight that sample size might not be the only relevant factor to increase the detection of such associations. From a more theoretical point of view, a dose effect of victimization should also be considered. More specifically, apart from large effects between general victimization vs. no bullying experiences, there might be some smaller associations between (only) the more intense or more frequently victimized and emotional intelligence characteristics. This calls for not only a general increase in the sample size but also a more diverse representation of victimization occurrences.

Previous studies which have examined emotion recognition accuracy of victims had considerably more participants (e.g., Woods et al., 2009) and some also more frequently bullied victims (e.g., Ciucci et al., 2014) and reported significant differences between victims and non-involved individuals. However, there is also one large study (Guy et al., 2017; frequency of victimization not apparent) that did not find victims to recognize emotions differently than non-involved peers. This discrepancy in findings in previous and in the present study warrants more research to establish whether there are indeed differences in emotion recognition between victims and non-involved individuals and how depression symptoms influence this association.

Contemporary integrative interpersonal theory (Pincus, 2005; Pincus and Ansell, 2013) proposes that interpersonal situations are a dynamic interplay of perceptions, behaviors, and affect. Therefore, similar to what we hypothesized, one could expect that a biased perception of others (as indicated by a potential hostile attribution bias) would also lead to an adjustment of behavioral responses, such as behaving more submissively when thinking others want to do harm. However, victims in our sample did not show a differential response to negative emotions compared to non-involved peers, or at least we were not able to detect such associations (see discussion of statistical power above). Previous research suggests that perceiving negative situations as uncontrollable and unchangeable is associated with processing negative stimuli more internally by exhibiting so-called characterological self-blame (cf. Graham and Juvonen, 1998). Engaging in characterological self-blame is associated with interpersonal and internalizing problems. Specifically in victims, high tendencies for characterological self-blame have been suggested to not only partly explain re- victimization (Schacter et al., 2015) but also victims’ depression symptoms (Perren et al., 2013). Whether victims in our sample also exhibited characterological self-blame remains to be determined.

Our results suggest that victims have similar cognitive empathy skills as non-bullied individuals. This potentially implies that having been bullied is not associated with an altered understanding of how others feel, at least not when it is about happy or sad content as in our task. It is worth noting however, that the entire sample performed poorly on the EAT compared to previous studies, specifically when watching the negative/sad videos. Research describes adolescence as a period characterized by a maturation of social and interpersonal competencies (Crone and Dahl, 2012). Notably, concepts such as cognitive empathy and theory of mind are said to develop until adulthood (e.g., Blakemore, 2012). Therefore, empathic accuracy skills of teenagers in our sample were possibly not as far developed. The task itself could have also influenced our participants’ performances. Participants might have had problems relating to the targets, possibly due to their age (mainly mid-twenties, i.e., 23–26years and one target with the age of 62) or content of the autobiographical events (see Materials and Methods). Therefore, we are uncertain to what degree our (non-significant) findings could be explained by participant’s cognitive maturity or the stimuli of the task itself.

We cannot say to what degree maturation of social and interpersonal competencies of our participants might have also potentially impacted the performance on the other two tasks. Though, regarding the tasks themselves, we did choose novel methodologies to assess interpersonal skills. VR is considered an appealing tool among adolescents and enabled us to assess emotion recognition in a more ecological valid and immersive manner as compared to some previous studies (Gutiérrez-Maldonado et al., 2014). In the present study, 62% of participants rated the VR-task as fairly or quite realistic and 28% as a little realistic (the remaining 10% as not realistic). These numbers are comparable to another study which applied the same VR task and in which the majority of participants also indicated that VR characters and their facial expressions looked realistic (cf. Nijman et al., 2020), which supports ecological validity of the task. Regarding the FERT, we assessed potential behavioral responses to others’ emotions while keeping a controlled and comparable environment. However, we are aware that indicating likely behavior based on a static photograph does not necessarily very well represent actual behavioral responses in real-life interpersonal encounters. Nevertheless, the FERT offers the assessment of likely interpersonal behaviors in response to facial emotions as compared to assessing behaviors more generally, without taking situational context such as the other person’s emotional state into account.

Interpersonal situations are complex. Understanding them requires skills to interpret verbal and non-verbal cues which can differ per emotion (cf. Hall et al., 2000). Emotions included in our tasks were intentionally mainly negative; however, the type and amount of emotions differed between tasks. For example, the ERT-VR and FERT did not include sadness, while the EAT mainly consisted of negative videos with sad content, and fearful stimuli were only included in the ERT-VR. This is because we used pre-set tasks and did not adjust them. Therefore, the comparability between tasks and emotions regarding social-emotional competencies is somewhat reduced.

Research has reported gender differences regarding emotional intelligence and competence in victims (Ciucci et al., 2014; van Noorden et al., 2017). Similarly, type and severity of victimization can also influence social-emotional skills (e.g., Woods et al., 2009; van Noorden et al., 2016). Due to small group sizes, we did not run additional tests including gender or victimization specific characteristics to prevent an increased false positive rate through a large number of tests. However, we did statistically control for underlying variation due to participants’ depression symptoms, which have also been shown to effect interpersonal skills (e.g., Dalili et al., 2015). While future studies should increase the sample size to test for additional influential effects, the time span of the assessed victimization should also be considered. Possibly, the association between victimization and social-emotional competencies is different for more recent (i.e., in the past month) compared to victimization that potentially happened some time ago (i.e., during high school, as in our study).

While we did choose tasks which were at parts more ecologically valid and immersive than previous studies to examine social and emotional competencies in victims, these computerized tasks cannot fully represent actual real-life interpersonal situations. As interpersonal situations are complex and situation-specific (Hopwood et al., 2019), researchers have suggested studying interpersonal processes with ecologically valid approaches such as ecological momentary assessment methodology (Reis, 2014). Specifically, event-contingent recording of social interactions can be used to examine the link between interpersonal perceptions and behavior (Moskowitz and Sadikaj, 2014). Therefore, future research examining interpersonal processes of victims would potentially benefit from applying such methodologies.

## Conclusion

We used novel methods to examine multiple aspects of emotional intelligence and emotional competence in victims of bullying and non-involved individuals and illustrated how these potentially contribute to interpersonal struggles of victims. Overall, the findings indicated that how victims perceive facial expressions, and how they potentially respond to facial expressions as well as their ability to understand how others feel were largely similar to non-involved individuals. Of note, as the present study only had sufficient power to detect large effects, generalization of our findings is limited and it cannot be ruled out that more subtle group differences remained undetected.

## Data Availability Statement

The data that support the findings of this study are available from the corresponding author, MF, upon reasonable request.

## Ethics Statement

The studies involving human participants were reviewed and approved by Ethics Committee of the Faculty of Behavioural and Social Sciences at the University of Groningen (EC-BSS). Written informed consent from the participants’ legal guardian/next of kin was not required to participate in this study in accordance with the national legislation and the institutional requirements.

## Author Contributions

MF conceived the study, participated in its design, coordinated and gathered data, performed statistical analyses, and drafted the manuscript. PJ participated in the design of the study and helped to draft the manuscript. WV provided parts of the measurement equipment, and helped to draft the manuscript. MR conceived the study, participated in its design, helped with statistical analyses and interpretation of the data, and drafted the manuscript. All authors contributed to the article and approved the submitted version.

## Funding

The present study was funded by a general PhD-research-grant of the Faculty of Behavioral and Social Sciences, University of Groningen (no grant number available).

## Conflict of Interest

The authors declare that the research was conducted in the absence of any commercial or financial relationships that could be construed as a potential conflict of interest.

## Publisher’s Note

All claims expressed in this article are solely those of the authors and do not necessarily represent those of their affiliated organizations, or those of the publisher, the editors and the reviewers. Any product that may be evaluated in this article, or claim that may be made by its manufacturer, is not guaranteed or endorsed by the publisher.
